# Coffee Consumption, Newly Diagnosed Diabetes, and Other Alterations in Glucose Homeostasis: A Cross-Sectional Analysis of the Longitudinal Study of Adult Health (ELSA-Brasil)

**DOI:** 10.1371/journal.pone.0126469

**Published:** 2015-05-15

**Authors:** James Yarmolinsky, Noel T. Mueller, Bruce B. Duncan, Maria del Carmen Bisi Molina, Alessandra C. Goulart, Maria Inês Schmidt

**Affiliations:** 1 Postgraduate Studies Program in Epidemiology, School of Medicine, Federal University of Rio Grande do Sul, Porto Alegre, Rio Grande do Sul, Brazil; 2 Department of Epidemiology, Mailman School of Public Health, Columbia University Medical Center, New York, NY, United States of America; 3 Institute of Human Nutrition and Department of Medicine, College of Physicians and Surgeons, Columbia University Medical Center, New York, NY, United States of America; 4 Center for Health Sciences, Federal University of Espirito Santo, Vitória, Espírito Santo, Brazil; 5 Center for Clinical and Epidemiological Research, Hospital Universitário, University of São Paulo, São Paulo, SP, Brazil; University of Miami School of Medicine, UNITED STATES

## Abstract

**Introduction:**

Observational studies have reported fairly consistent inverse associations between coffee consumption and risk of type 2 diabetes, but this association has been little investigated with regard to lesser degrees of hyperglycemia and other alterations in glucose homeostasis. Additionally, the association between coffee consumption and diabetes has been rarely investigated in South American populations. We examined the cross-sectional relationships of coffee intake with newly diagnosed diabetes and measures of glucose homeostasis, insulin sensitivity, and insulin secretion, in a large Brazilian cohort of middle-aged and elderly individuals.

**Methods:**

We used baseline data from 12,586 participants of the Longitudinal Study of Adult Health (ELSA-Brasil). Logistic regression analyses were performed to examine associations between coffee consumption and newly diagnosed diabetes. Analysis of covariance was used to assess coffee intake in relation to two-hour glucose from an oral glucose tolerance test, fasting glucose, glycated hemoglobin, fasting and –2-hour postload insulin and measures of insulin sensitivity.

**Results:**

We found an inverse association between coffee consumption and newly diagnosed diabetes, after adjusting for multiple covariates [23% and 26% lower odds of diabetes for those consuming coffee 2–3 and >3 times per day, respectively, compared to those reporting never or almost never consuming coffee, (p = .02)]. An inverse association was also found for 2-hour postload glucose [Never/almost never: 7.57 mmol/L, ≤1 time/day: 7.48 mmol/L, 2-3 times/day: 7.22 mmol/L, >3 times/day: 7.12 mol/L, p<0.0001] but not with fasting glucose concentrations (p = 0.07). Coffee was additionally associated with 2-hour postload insulin [Never/almost never: 287.2 pmol/L, ≤1 time/day: 280.1 pmol/L, 2–3 times/day: 275.3 pmol/L, >3 times/day: 262.2 pmol/L, p = 0.0005) but not with fasting insulin concentrations (p = .58).

**Conclusion:**

Our present study provides further evidence of a protective effect of coffee on risk of adult-onset diabetes. This effect appears to act primarily, if not exclusively, through postprandial, as opposed to fasting, glucose homeostasis.

## Introduction

Over the past decade there has been an emergence of literature on the association between coffee intake and type 2 diabetes, and a recent meta-analysis of studies on this topic concluded that regular intake of caffeinated and decaffeinated coffee is inversely associated with risk of type 2 diabetes in a dose-dependent manner [1].Yet, the preponderance of studies in this literature derives from high income countries where the confounding structures related to coffee intake and diabetes may differ from those of low-middle income countries. Moreover, few epidemiological studies—and almost none in the less developed world—have reported on coffee intake in relation to the various measures of glucose and insulin homeostasis. These are of particular interest as they provide insight into the key physiological aspects of glucose metabolism affected by coffee intake. Those that have been conducted in high income countries suggest that coffee consumption may affect postprandial blood glucose more strongly than fasting glucose concentrations [2–6]. There is less consistent evidence for whether coffee is more strongly associated with insulin resistance or pancreatic beta-cell function [2,4]. Lastly, no studies have evaluated the effect of coffee intake on postprandial metabolism of glucose in individuals with diabetes, after a meal test.

The Brazilian Longitudinal Study of Adult Health (ELSA-Brasil), a prospective cohort investigation of 15,105 Brazilian Adults from various regions of the country, provides a unique opportunity to study the association of coffee intake with diabetes and measures of glucose and insulin homeostasis. To date, no known studies have investigated an association between coffee and clinically measured diabetes in Brazil and elsewhere in South America. The extensive laboratory measurements performed in ELSA-Brasil, including the use of a standardized post-meal test and postprandial samples for those with known diabetes and an oral glucose tolerance test (OGTT) with stored postload samples and measurements of glycated hemoglobin (HbA1c) for all other participants, permits robust investigations into various physiological aspects of glucose metabolism associated with coffee intake.

We evaluated cross-sectional associations between coffee intake, newly diagnosed diabetes, fasting plasma glucose, two-hour plasma glucose during a 75g oral-glucose tolerance test, glycated hemoglobin, and measures of insulin resistance and beta-cell secretion. We also analyzed the relationship between coffee intake and glucose/insulin metabolism in participants with previously diagnosed diabetes during a standardized meal test.

## Methods

### Study design and population

The Longitudinal Study of Adult Health (In Portuguese, Estudo Longitudinal de Saude do Adulto or ELSA-Brasil) is a cohort study designed to identify risk factors for diabetes and cardiovascular disease. The details of the study, including design, eligibility criteria, sources and methods of recruitment, and measurements obtained, have been described in detail elsewhere [7]. The cohort comprises 15,105 civil servants, aged 35 to 74 years at baseline (2008–2010), who were sampled from universities or research institutions located in six cities of three different regions of Brazil. All data for the current cross-sectional analyses were collected at baseline during initial interviews (~1 hour) and the first clinic visit (~5 hours). The study was approved by the Research and Ethics Committees of the institutions involved: Hospital de Clinicas de Porto Alegre, Universidade Federal do Rio Grande do Sul, Universidade Federal do Espírito Santo, Universidade Federal de Minas Gerais, Universidade Federal da Bahia, Universidade de São Paulo, Fundação Osvaldo Cruz. All participants provided written, informed consent for their clinical records to be used in this study prior to enrolment.

For the current investigation, we excluded participants with missing data at baseline for the following variables: coffee intake (n = 28), body mass index (n = 6), waist-hip ratio (n = 8), race/color (n = 184), education of mother (n = 366), leisure time physical activity (n = 221), alcohol intake (n = 26), various dietary variables (n = 72), hypertension (n = 13), and laboratory measurements (n = 352). Some participants had missing data for two or more variables. We further excluded those with previously diagnosed diabetes (n = 1473) from all main analyses, except for those of coffee intake and glucose concentrations after a two-hour post-meal test. Consequently, our main analyses were performed on 12,586 participants. Analyses of coffee consumption with 2-hour post-meal glucose among those with previously diagnosed diabetes were performed on 1,077 participants.

### Exposure assessment

Coffee intake was assessed at the baseline examination through a validated 114-item Food Frequency Questionnaire (FFQ) [8]. Reproducibility and relative validity of the FFQ compared to three food records completed by a sub-set of participants and assessed through the intra-class correlation coefficient (ICC) was shown to have a reproducibility ranging from 0.55 to 0.83 and a relative validity ranging from 0.20 to 0.72, depending on the nutrient value. Participants were asked to provide information on their typical eating habits in the twelve months prior to the baseline examination of the ELSA-Brasil. For assessment of habitual coffee intake, specifically, participants were asked to provide both the typical frequency with which they consumed coffee (>3 times/day, 2–3 times/day, 1 time/day, 5–6 times/week, 2–4 times/week, 1 time/week, 1–3 times/month, never/almost never) and the typical quantity of coffee consumed on each occasion in relation to a reference cup size of 50 mL. Participants were further asked to specify the type of coffee normally consumed (filter, instant, espresso, moka pot), whether this coffee contained caffeine (caffeinated or decaffeinated), and whether additional items were typically added to the coffee (sugar, artificial sweetener, none).

### Outcome ascertainment

A 12-hour fasting blood sample was drawn by venipuncture soon after each participant arrived at the baseline clinic visit. A two-hour 75g OGTT was then administered to participants without known diabetes. A two-hour post-meal test, consisting of 47 g of rapidly digested carbohydrates and 14 g of saturated fat from dairy products, was administered to those with known diabetes. Glucose was measured centrally by the hexokinase method (ADVIA Chemistry; Siemens, Deerfield, Illinois), percent glycated hemoglobin (A1C) was measured using a high-pressure liquid chromatography (Bio-Rad Laboratories, Hercules, California), a National Glycohemoglobin Standardization Program (NGSP) certified method, and insulin was measured with an immunoenzymatic assay (ELISA) (Siemens).

Diabetes status was classified using blood glucose measurements and self-reported information. A participant was considered to have previously diagnosed diabetes when answering, “yes” to either “Have you been previously told by a physician that you had diabetes (sugar in the blood)?” or “Have you used medication for diabetes in the past 2 weeks?” The remaining participants were evaluated for undiagnosed diabetes based on their laboratory values and then classified as having diabetes if they reached the threshold for fasting plasma glucose (≥ 7.0 mmol/L), 2-hour postload plasma glucose (≥ 11.1 mmol/L), or HbA1c (≥ 6.5%) [9,10]. Given the age range of our participants, the vast majority of the previously undiagnosed cases of diabetes will have type 2 diabetes [11].

Impaired glucose tolerance was defined as a two-hour postload glucose concentration ≥7.8 mmol/L and <11.1 mmol/L; impaired fasting glucose was defined as a fasting glucose concentration ≥6.1 mmol/L and <7.0 mmol/L [10,12]; and elevated HbA1c by a value ≥ 5.7%, <6.5% [12]. Elevated fasting and two-hour postload insulin were defined as the top quartile of their respective distribution of values, as follows: elevated fasting insulin ≥ 61.8 pmol/L, and elevated two-hour postload insulin ≥447.0 pmol/L. In participants with previously known diabetes, outcome of interest was elevated two-hour postprandial glucose (≥14.87 mmol/L, the top quartile of its distribution).

### Covariate assessment

A comprehensive set of questionnaires, tests, and measurements [13] was carried out. To control for confounding, we selected established risk factors for diabetes and factors known to be associated with coffee consumption based on prior studies and theoretical considerations as follows: age at baseline visit (years), race/skin color, educational achievement of the participant and their mother, smoking status, alcohol use, physical activity (ascertained using the leisure time section of the long version of the International Physical Activity Questionnaire [14]), family history of diabetes, hypertension, and various dietary variables were ascertained by questionnaire, and blood pressure was measured using an oscillametric method (Omron HEM 705CP) [15]. Body mass index (BMI) was calculated as weight (kg) divided by height (m) squared. The hepatic enzymes alanine aminotransferase and aspartate aminotransferase were measured centrally by the Modified International Federation for Clinical Chemistry (enzymatic) assay (ADVIA Chemistry) and gamma-glutamyltransferase was measured by the kinetic calorimetric assay.

### Statistical analyses

Baseline characteristics of the sample were summarized using unadjusted means and standard deviations for continuous variables and frequencies and percentages for categorical variables according to coffee intake category (Never/almost never [less than 1 time per month; referent category], at least 1 time per month but no greater than 1 time per day, 2–3 times per day, >3 times per day). As quantity of coffee typically consumed on each occasion did not differ markedly by frequency of coffee consumption, analyses were performed by frequency of coffee consumption alone.

Analysis of covariance (ANCOVA) was performed to assess the association between category of coffee intake and markers of glucose homeostasis as continuous variables. In participants without previously known diabetes, outcomes of interest were fasting plasma glucose, 2-hour postload plasma glucose, HbA1c, fasting insulin, two-hour postload insulin, homeostasis model assessment of insulin resistance (HOMA-IR) and of β-cell function (HOMA-β), and the insulin sensitivity index (ISI-composite) proposed by DeFronzo and Matsuda [16]. In participants with known diabetes who underwent the meal challenge test, outcome of interest was two-hour postprandial glucose. Insulin, HOMA-IR, HOMA-β, and ISI-composite measures were log-transformed then converted to geometric means. Multivariable logistic regression was performed to generate odds ratios (ORs) and 95% confidence intervals for the association between category of coffee intake and diabetes and categorized markers of glucose homeostasis. In participants without previously known diabetes, outcomes of interest were adult-onset diabetes, impaired fasting glucose, impaired glucose tolerance, HbA1c ≥ 5.7%, <6.5%, elevated fasting insulin, and elevated two-hour postload insulin. For impaired glucose tolerance, impaired fasting glucose, and HbA1c ≥ 5.7%, <6.5% analyses, participants with newly diagnosed diabetes were further excluded. Consequently, these analyses were performed on 11,245 participants.

Interaction terms were used to investigate whether associations differed between the following pre-specified sub-groups: sex, age (median cut-point; ˂51 years, ≥51 years), race/skin color (white, non-white), BMI (<25 kg/m^2^, ≥25 kg/m^2^), and smoking status (current, past, never smoker). All statistical tests were two-sided and significance was defined at p<0.05. Statistical analyses were performed with SAS 9.4 (SAS Institute, Inc., Cary, North Carolina).

## Results

Analyses were initially performed separately by method of coffee preparation (filtered, instant, moka pot, espresso), type of coffee (caffeinated or decaffeinated), and addition of sweetener to coffee (sugar, alternative sweetener, none) (S1 Table). As resulting sample sizes were frequently small and there were no statistically significant differences across coffee preparation methods, types of coffee, or addition of sweetener to coffee in relation to our main outcomes, all additional analyses were performed without consideration of these characteristics.

Baseline descriptive data of ELSA-Brasil participants by category of coffee intake are presented in Table 1. Most participants (around 58%) reported consuming coffee at least twice per day, with 2928 (around 23% of the total) reporting consuming coffee more than 3 times per day. Most participants reported typically consuming coffee that was filtered (83%), caffeinated (98%), and with added sugar or artificial sweetener (59% and 28%, respectively). Participants who reported greater daily coffee intake were more likely to consume alcohol and be current smokers than those who reported less frequent coffee intake.

**Table 1:** Characterization of ELSA-Brasil participants (N = 12586) by frequency of coffee consumption per day, at baseline (2008–2010).

Variables	Total	Never/ almost never	≤1	2–3	>3
	(N = 12586)	(n = 1235)	(n = 4044)	(n = 4379)	(n = 2928)
Male (%)	5663 (45.0%)	587 (47.5%)	1808 (44.7%)	1774 (40.5%)	1494 (51.0%)
Age, years (SD)	51.4 (8.9)	50.0 (9.1)	51.6 (9.3)	52.1 (9.1)	50.7 (7.8)
Race/color					
Black	1912 (15.2%)	263 (21.3%)	675 (16.7%)	595 (13.6%)	379 (12.9%)
Brown (‘Pardo’)	3488 (27.7%)	346 (28.0%)	1098 (27.1%)	1237 (28.2%)	807 (27.6%)
White	6776 (53.8%)	587 (47.5%)	2122 (52.5%)	2412 (55.1%)	1655 (56.5%)
Other^a^	410 (3.3%)	39 (3.2%)	149 (3.7%)	135 (3.1%)	87 (3.0%)
Low educational attainment^b^ (%)	4794 (38.1%)	457 (37.0%)	1578 (39.0%)	1648 (37.6%)	1111 (37.9%)
BMI, kg/m^2^ (SD)	26.8 (4.6)	26.8 (5.0)	26.8 (4.6)	26.7(4.6)	26.8 (4.6)
WHR (SD)	.89 (.09)	.89 (.09)	.89 (.09)	.88 (.09)	.89 (.09)
Current smoker (%)	1640 (13.0%)	76 (6.2%)	378 (9.4%)	436 (10.0%)	750 (25.6%)
Alcohol user (%)	8927 (70.9%)	713 (57.7%)	2863 (70.8%)	3143 (71.8%)	2208 (75.4%)
Low physical activity^c^ (%)	10460 (83.1%)	1028 (83.2%)	3313 (81.9%)	3616 (82.6%)	2505 (85.6%)
Tea, g/day (SD)	46.7 (92.3)	46.5 (93.8)	44.4 (89.6)	46.9 (92.8)	49.6 (94.2)
Hypertension (%)	4064 (32.3%)	362 (29.3%)	1398 (34.6%)	1481 (33.8%)	823 (28.1%)
Family history of diabetes (%)	4584 (36.4%)	443 (35.9%)	1441 (35.6%)	1635 (37.3%)	1065 (36.4%)

Values are means and standard deviations for continuous variables and frequencies and percentages for categorical variables. Abbreviations: BMI, body mass index (kg/m^2^); WHR, waist-hip ratio.

^a^ “Other”: those reporting their race/skin color as “Asian” or “Indigenous.

^b^ Highest level of instruction completed was High school, or less.

^c^ Typically engage in leisure time physical activity 1 day/week or less.

At baseline, we found 1341 (10.7%) cases of newly-diagnosed diabetes, 3083 (24.5%) cases of impaired fasting glucose, 3114 (24.7%) cases of impaired glucose tolerance, and 2651 (21.1%) cases of HbA1c ≥ 5.7%, <6.5%.

**Fig 1:**
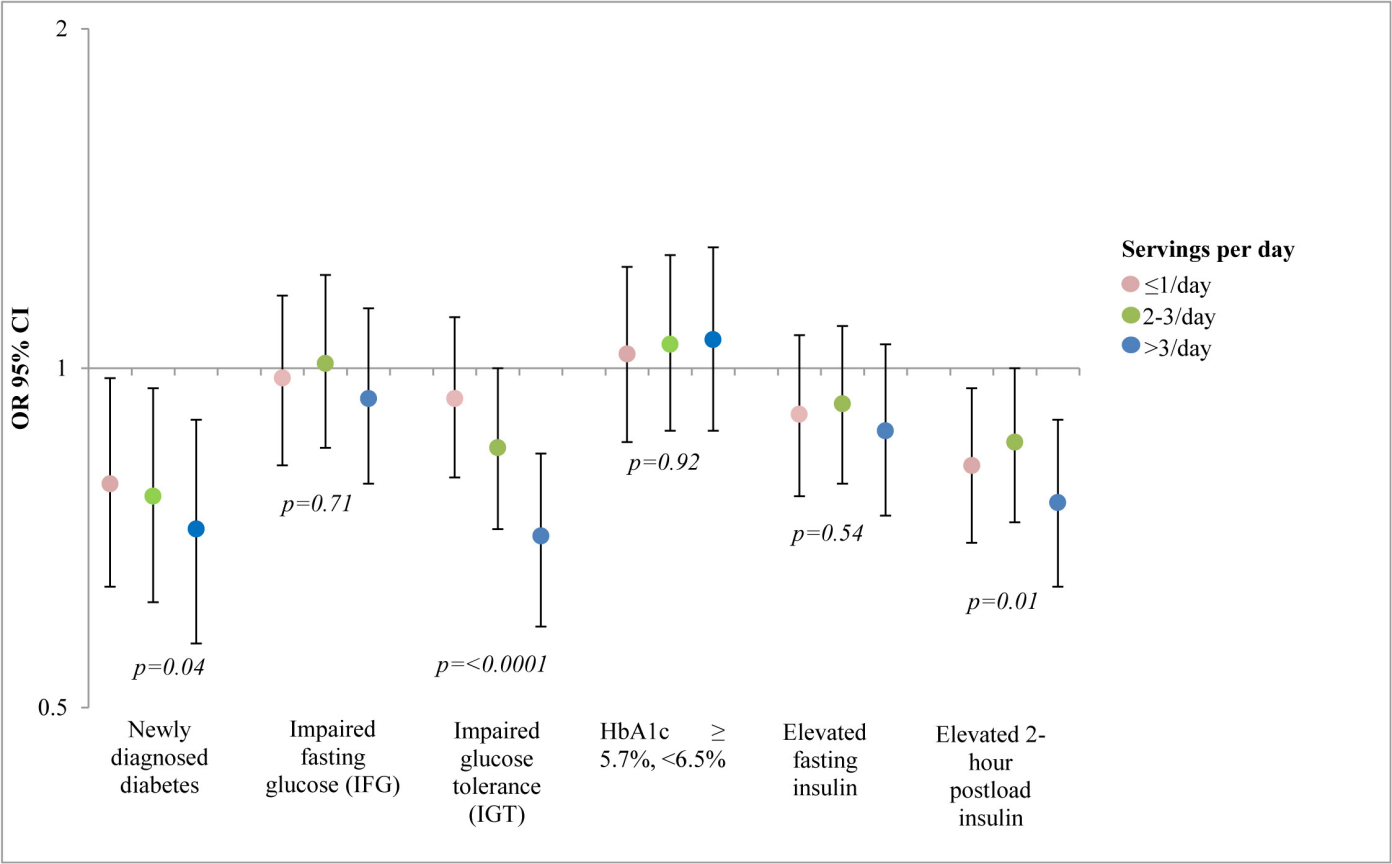
Fully-adjusted (Model 3) associations of frequency of coffee consumption per day with newly diagnosed diabetes and intermediate hyperglycemia, from ELSA-Brasil (2008–2012) (N = 12586). For IFG, IGT, HbA1c analyses, n = 11245 after exclusion of participants with newly diagnosed diabetes.

We first present analyses of outcomes for the different categories of hyperglycemia and insulinemia, followed by analyses of the laboratory variables and their HOMA derivatives when considered in their continuous form. While infrequent or moderate (≤1 per day) consumption of coffee was not consistently associated with glucose outcome measures, participants who consumed coffee 2–3 and >3 times per day had a 23% (6–37%) and 26% (8–40%) lower adjusted odds of newly diagnosed diabetes, respectively, compared to those who never consumed coffee (P = 0.02) (Model 1, Table 2). Participants reporting consuming coffee >3 times per day also had a 29% (16–40%) lower adjusted odds of impaired glucose tolerance (P<0.0001). Lastly, those consuming coffee both 2–3 and >3 times per day reported lower adjusted odds of elevated 2-hour postload insulin, compared to those who never consumed coffee (P = 0.002). These associations did not change appreciably when we adjusted for hypertension medication use or menopausal status (not shown) and remained significant after full covariate adjustment (Model 3, Fig 1). Slight attenuation was seen in Model 4, which added potential mediators. There were no appreciable associations between these higher coffee intake categories and impaired fasting glucose, HbA1c ≥ 5.7%, <6.5%, or elevated fasting insulin (Table 2).

**Table 2:** Adjusted^*^ associations of frequency of coffee consumption per day with newly diagnosed diabetes, intermediate hyperglycemia, and insulin levels, from ELSA-Brasil (2008–2012) (N = 12586).

	Never/almost never	≤1/day	2-3/day	>3/day	P-value†
	(n = 1235)	(n = 4044)	(n = 4379)	(n = 2928)	
	OR (95% CI)	OR (95% CI)	OR (95% CI)	OR (95% CI)	
**Newly diagnosed diabetes**					
Model 1	1.00	.85 (.69–1.04)	.77 (.63-.94)	.74 (.60-.92)	.02
Model 2	1.00	.82 (.66–1.01)	.77 (.62-.94)	.73 (.58-.91)	.04
Model 3	1.00	.79 (.64-.98)	.77 (.62-.96)	.72 (.57-.90)	.04
Model 4	1.00	.80 (.64-.99)	.77 (.62-.95)	.73 (.58-.92)	.047
**Impaired fasting glucose (IFG)** ^a^					
Model 1	1.00	1.05 (.89–1.24)	1.06 (.90–1.26)	1.01 (.85–1.21)	.81
Model 2	1.00	.99 (.84–1.18)	1.01 (.85–1.20)	.96 (.80–1.15)	.89
Model 3	1.00	.98 (.82–1.16)	1.01 (.85–1.21)	.94 (.79–1.13)	.71
Model 4	1.00	.99 (.83–1.18)	1.03 (.86–1.22)	.96 (.80–1.15)	.75
**Impaired glucose tolerance (IGT)** ^a^					
Model 1	1.00	.99 (.85–1.16)	.86 (.74–1.01)	.71 (.60-.84)	<0.0001
Model 2	1.00	.97 (.83–1.14)	.85 (.72–1.00)	.73 (.61-.87)	<0.0001
Model 3	1.00	.94 (.80–1.11)	.85 (.72–1.00)	.71 (.59-.84)	<0.0001
Model 4	1.00	.97 (.81–1.16)	.87 (.73–1.04)	.74 (.61-.90)	.001
**HbA1c ≥ 5.7%, <6.5%** ^a^					
Model 1	1.00	1.00 (.84–1.20)	.99 (.83–1.18)	1.06 (.89–1.27)	.73
Model 2	1.00	1.03 (.86–1.23)	1.05 (.88–1.25)	1.06 (.88–1.28)	.93
Model 3	1.00	1.03 (.86–1.23)	1.05 (.88–1.26)	1.06 (.88–1.28)	.92
Model 4	1.00	1.03 (.86–1.23)	1.06 (.89–1.26)	1.07 (.89–1.29)	.88
**Elevated fasting insulin** ^b^					
Model 1	1.00	.96 (.83–1.12)	.92 (.79–1.06)	.87 (.75–1.02)	.23
Model 2	1.00	.94 (.81–1.09)	.91 (.78–1.05)	.91 (.78–1.07)	.59
Model 3	1.00	.91 (.77–1.07)	.93 (.79–1.09)	.88 (.74–1.05)	.54
Model 4	1.00	.91 (.77–1.07)	.93 (.79–1.09)	.88 (.74–1.05)	.55
**Elevated 2-hour postload insulin** ^c^					
Model 1	1.00	.86 (.75–1.00)	.85 (.74-.98)	.75 (.64-.87)	.002
Model 2	1.00	.87 (.75–1.00)	.86 (.74-.99)	.80 (.68-.93)	.0496
Model 3	1.00	.82 (.70-.96)	.86 (.73–1.00)	.76 (.64-.90)	.01
Model 4	1.00	.82 (.70-.96)	.86 (.74–1.00)	.76 (.64-.89)	.01

^a^ For IFG, IGT, HbA1c analyses, n = 11245 after exclusion of participants with newly diagnosed diabetes.

^b^ ≥ 61.8 pmol/l (top quartile of distribution).

^c^ ≥ 447.0 pmol/l (top quartile of distribution).

† P-value for the test of any association between coffee consumption and the outcome of interest

* Model 1: adjusted for sex, age (years), ELSA-Brasil center.

Model 2: + race/color (white, pardo, black, asian/indigenous), education (high school or less, some university or more), education of mother (high school or less, some university or more), smoking status (current, former, never smoker), alcohol intake (user, former user, never user), leisure time physical activity level (engage in physical activity one time per week or less, engage in physical activity two or more times per week), hypertension, family history of diabetes, daily fruit consumption, daily vegetable consumption, dairy product intake (g/day), beef intake (g/day), white rice intake (g/day), soda intake (g/day), juice intake (g/day), tea intake (g/day), % kcal from fat

Model 3: + body mass index, waist-hip ratio, C-reactive protein.

Model 4: + Magnesium. Further adjustment for insulin measures (fasting and 2-hour postload) for diabetes, fasting glucose, two-hour postload glucose, and HbA1c analyses.

When we analyzed associations between coffee intake and these outcomes in a continuous fashion, thus describing the whole spectrum of glucose regulation, from normal to intermediate states and diabetes, similar associations were observed (Table 3). Further, we found a significant association of coffee intake with mean ISI-composite, which was maintained in our fully-adjusted model. There was no evidence for interaction by sex, age, race/skin color, BMI, or smoking status.

**Table 3:** Adjusted^*^ Mean (SE) values for metabolic measures according to the relationship with frequency of coffee consumption per day. ELSA-Brasil, 2008–2012 (N = 12586).

	Never/almost never	≤1	2–3	>3	P-value†
	(n = 1235)	(n = 4044)	(n = 4379)	(n = 2928)	
**Fasting glucose (mmol/L)**					
Model 1	5.95±0.03	5.96±0.02	5.91±0.01	5.92±0.02	.07
Model 2	5.99±0.04	5.98±0.03	5.94±0.03	5.95±0.03	.10
Model 3	5.98±0.04	5.97±0.03	5.94±0.03	5.94±0.03	.22
Model 4	5.99±0.04	5.97±0.03	5.93±0.03	5.93±0.03	.08
**Two-hour postload glucose (mmol/L)**					
Model 1	7.57±0.07	7.48±0.04	7.22±0.04	7.12±0.05	<0.0001
Model 2	7.60±0.10	7.47±0.08	7.24±0.08	7.18±0.09	<0.0001
Model 3	7.57±0.10	7.41±0.08	7.22±0.08	7.13±0.08	<0.0001
Model 4	7.57±0.09	7.44±0.08	7.26±0.08	7.20±0.08	<0.0001
**HbA1c (%)**					
Model 1	5.3±0.02	5.3±0.01	5.3±0.01	5.3±0.01	.62
Model 2	5.4±0.03	5.4±0.02	5.4±0.02	5.4±0.02	.75
Model 3	5.4±0.03	5.4±0.02	5.4±0.02	5.4±0.02	.62
Model 4	5.4±0.03	5.4±0.02	5.4±0.02	5.4±0.02	.61
**Fasting insulin (pmol/L)**					
Model 1	32.8±0.8	33.1±0.5	33.4±0.5	32.4±0.6	.58
Model 2	31.9±1.1	32.0±0.9	32.4±1.0	32.3±1.0	.87
Model 3	31.8±1.0	31.3±0.8	32.5±0.8	31.6±0.8	.10
Model 4	31.8±1.0	31.3±0.8	32.5±0.8	31.6±0.8	.10
**Two-hour postload insulin (pmol/L)**					
Model 1	287.2±6.4	280.1±3.5	275.3±3.3	262.2±3.9	.0005
Model 2	272.3±8.5	265.9±6.9	261.9±6.8	256.2±6.8	.08
Model 3	269.4±7.8	259.6±6.2	260.9±6.3	250.9±6.3	.02
Model 4	269.4±7.8	259.6±6.3	260.9±6.3	250.9±6.3	.02
**HOMA-IR**					
Model 1	1.43±0.04	1.45±0.02	1.45±0.02	1.41±0.03	.58
Model 2	1.40±0.05	1.40±0.04	1.42±0.04	1.41±0.05	.97
Model 3	1.39 ±0.04	1.37±0.04	1.42±0.04	1.38±0.04	.16
Model 4	1.39±0.04	1.37±0.04	1.42±0.04	1.38±0.04	.16
**HOMA-β**					
Model 1	48.1 ±0.5	47.5±.3	48.4±.3	48.2±.3	.14
Model 2	46.8 ±0.7	46.7±.6	47.4±.6	47.3±.6	.17
Model 3	47.0 ±0.7	47.0±.6	47.5±.6	47.5±.6	.33
Model 4	47.0 ±0.7	47.0±.6	47.5±.6	47.5±.6	.32
**ISI (composite)**					
Model 1	5.28 ±0.13	5.34±.07	5.47±.07	5.73±.09	.003
Model 2	5.47 ±0.18	5.57±.15	5.67±.16	5.77±.17	.17
Model 3	5.53 ±0.16	5.74±.14	5.68±.14	5.92±.15	.02
Model 4	5.54 ±0.16	5.72±.13	5.63±.13	5.88±.14	.02

Values presented are Mean ± SE (Standard error). Fasting insulin, two-hour postload insulin, HOMA-IR, HOMA-β, & ISI (composite) are presented as Geometric means. To convert to conventional units: mmol*18.018 = mg/dl; pmol/l*.167 = μIU/ml.

† P-value for the test of any association between coffee consumption and the outcome of interest

* Model 1: adjusted for sex, age (years), ELSA-Brasil center.

Model 2: + race/color (white, pardo, black, asian/indigenous), education (high school or less, some university or more), education of mother (high school or less, some university or more), smoking status (current, former, never smoker), alcohol intake (user, former user, never user), leisure time physical activity level (engage in physical activity one time per week or less, engage in physical activity two or more times per week), hypertension, family history of diabetes, daily fruit consumption, daily vegetable consumption, dairy product intake (g/day), beef intake (g/day), white rice intake (g/day), soda intake (g/day), juice intake (g/day), tea intake (g/day), % kcal from fat.

Model 3: + body mass index, waist-hip ratio, C-reactive protein.

Model 4: + Magnesium. Further adjustment for insulin measures (fasting and 2-hour postload) for fasting glucose, two-hour postload glucose, and HbA1c analyses.

Finally, when we added the three enzyme measures of hepatic function, a further potential mediator of the coffee-diabetes relationship, in an additional model, measures of association with individual consumption frequencies hardly changed (OR [95% CI] 0.80 [.64-.99], 0.78 [.63-.97], 0.74 [.59-.94] for ≤1/day, 2-3/day and >3/day, respectively, P for the overall association = 0.08). The association with two-hour postload glucose also changed little: 7.56±.09, 7.43±.08, 7.27±.08, and 7.20±.08 mmol/L for the categories never/almost never, ≤1/day, 2-3/day, and >3/day, respectively; P for the overall association <0.0001)

In analyses on participants with known diabetes, no associations were found between frequency of coffee consumption and elevated two-hour post-prandial glucose (OR [95% CI]: ≤1/day: .81 [.46–1.41]; 2-3/day: .90 [.52–1.56]; >3/day: 1.06 [.60–1.86] vs. Never/almost never [referent]; p-value = .57) or two-hour postprandial glucose (Mean [SE] mmol/l: Never/almost never: 11.9 [0.6]; ≤1/day: 12.0 [0.3]; 2-3/day: 12.0 [0.3]; >3/day: 12.3 [0.3]; p-value: .85). Analyses performed with coffee consumption categorized by quantity consumed instead of by frequency produced similar results (S2 Table).

## Discussion

In this cross-sectional analysis of 12,586 Brazilian adults 35–74 years of age who had not been previously diagnosed with diabetes, we found an association between higher intake of coffee (two or more times per day) and lower odds of newly diagnosed diabetes—most likely type 2—assessed at the baseline clinical examination. This association appeared to be primarily driven by impaired glucose and insulin regulation after a glucose challenge. We did not observe associations between coffee and glycated hemoglobin levels. We also did not find associations between coffee and measures of glucose regulation during a two hour post-meal test in participants with previously diagnosed diabetes.

This is the first comprehensive assessment of the association between coffee intake and glucose regulation conducted in a Brazilian population. Our findings extend beyond those of Machado et al. [17] which addressed the association only in those with self-reported diabetes.

Our finding of overall stronger inverse associations between coffee and impaired glucose tolerance, as compared with impaired fasting glucose, is consistent with previous studies [3,6], and provides further confirmation that the apparent protective effect of coffee consumption acts primarily through postprandial glucose homeostasis. We also noted a significant inverse association between coffee and postload insulin concentrations, but no associations with other markers of insulin resistance in the fasting state: HOMA-IR and fasting insulin concentrations. We did not observe associations of coffee with HOMA-β concentrations, a measure of insulin secretion, but did find an association with ISI composite, which aggregates fasting and postload insulin and glucose values.

There has been considerable inconsistency in analyses examining the effect of coffee on measures of both insulin resistance and secretion. In a cross-sectional analysis of 1,440 Japanese adults, higher coffee consumption was associated with decreased insulin resistance, but not with insulin secretion, as evaluated through the homeostatic model assessment [18]. Likewise, in a study of 1,088 elderly Swedish men, habitual coffee consumption was associated with increased insulin sensitivity as measured using a hyperinsulinemic-euglycemic clamp, but not with early insulin response during an OGTT [19]. However, in a separate Swedish study, higher coffee consumption was associated with a reduced risk of insulin resistance in women, and with low β-cell function in women with impaired glucose tolerance [4]. In a cross-sectional study of 954 multi-ethnic non-diabetic adults from the Insulin Resistance Atherosclerosis Study, Masters et al. (2012) reported stronger associations between caffeinated coffee and insulin sensitivity, while decaffeinated coffee was favorably related to measures of beta cell function, as assessed through frequently sampled intravenous glucose tolerance tests [2]. As noted in their analysis, few studies to date appear to have examined whether participants reporting regular coffee consumption tend to consume caffeinated as opposed to decaffeinated coffee and what potential differential effects these beverages may have on insulin activity. Separate analyses of the effects of caffeinated and decaffeinated coffee on all insulin measures in our study overlapped. However, as only two percent of our participants reported consuming decaffeinated coffee, we may have lacked the statistical power to detect a differential association. Mechanistic research is warranted to elucidate the extent to which coffee may enhance insulin secretion.

The lack of an association found between coffee intake and two hour postprandial glucose among participants with previously diagnosed diabetes is difficult to interpret as antidiabetic medication usage may well have masked any relationship of coffee with postprandial glucose levels. Additionally, knowledge of diabetes status may have modified dietary intake, including coffee consumption.

Major components of coffee include caffeine, chlorogenic acid, magnesium, adiponectin, quinides, and various other micronutrients. Several mechanisms have been proposed to explain the apparent protective effect of coffee consumption on type 2 diabetes. Although caffeine was originally considered to be the primary contributor to coffee’s effect on glucose metabolism, various studies reporting protective effects of decaffeinated coffee on diabetes now appear to suggest that other components of coffee may be more responsible for its antidiabetic effect. Magnesium has been proposed as a possible contributor to glucose metabolism by modulating insulin receptor activity through effects on hormone-receptor affinity or membrane viscosity [20]. We found that adjusting for magnesium intake in all of our analyses did not attenuate associations. This is consistent with more recent reports on this topic [21, 22]. Because coffee is a rich source of anti-oxidants, and sub-clinical inflammation has been implicated in the development of insulin resistance, it was proposed that coffee may improve insulin sensitivity by decreasing inflammation. An eight week randomized controlled trial found favourable effects of coffee intake on adiponectin concentrations which, aside from anti-inflammatory actions, act through AMPK (5' adenosine monophosphate-activated protein kinase) to modulate glucose uptake which may contribute to beneficial metabolic effects of longer term coffee intake [23]. Similarly, a recent trial showed a positive correlation between changes in caffeine level, a biomarker of coffee ingestion, and changes in adiponectin level when comparing zero versus 8 cups/day of coffee intake [24]. However, a cross-sectional study exploring the extent to which adiponectin and the inflammatory marker C-reactive protein (CRP) explained the association between regular coffee consumption and diabetes did not find evidence that this effect is mediated by anti-inflammatory mechanisms [5]. Likewise, adjusting for C-reactive protein in our analyses did not substantially weaken the observed associations. Chlorogenic acid, the second most abundant component of coffee, is also believed to play a role in reducing postprandial plasma glucose [25]. It may reduce hepatic glucose output through inhibition of glucose-6-phosphatase activity, may attenuate glucose absorption rates by inhibiting the sodium-dependent glucose transporters, and may reduce oxidative stress through its antioxidant properties [26]. Furthermore, it has been demonstrated that chlorogenic acid stimulates glucose transport in skeletal muscle through activation of AMPK [27]. Additionally, the roasting of coffee transforms some chlorogenic acid into quinides, compounds that have been shown to enhance insulin sensitivity in rats [28]. Whether chlorogenic acid mediates the observed associations requires further study. It has further been suggested that coffee may contribute to improvements in glucose homeostasis through the stimulation of incretins, gastrointestinal hormones responsible for regulation of postprandial insulin and glucagon production by the pancreas [29]. For example, two experimental studies in humans reported that caffeinated and decaffeinated coffee acutely decreased concentrations of glucose-dependent insulinotropic peptide (GIP) [30, 31], with one of the studies additionally reporting that decaffeinated coffee acutely increased concentrations of glucagon-like peptide 1 (GLP-1) [30]. However, a more recent study failed to find an effect of decaffeinated coffee on either GIP or GLP-1 responses during an oral glucose tolerance test [32]. A stimulatory effect of coffee on activity of incretins would be consistent with our finding of a stronger association of coffee with postprandial, as opposed to fasting, glucose concentrations. Lastly, the role of sex hormone binding globulin (SHBG) as a potential mediator between caffeinated coffee intake and reduced risk of diabetes among postmenopausal women has been proposed [33].

Some of the strengths of our study include the use of a standardized post-meal test and postprandial samples for those with known diabetes and a standard 75 g-OGTT with stored postload samples and measurements of glycated hemoglobin for all other participants. Previously unknown diabetes was determined on the basis of blood glucose measurements. Through collecting data on methods of coffee preparation, types of coffee typically consumed, and addition of sweetener, we were able to investigate potential differential effects on outcomes of interest. Another strength of this present analysis is its relatively large size—to our knowledge, the largest study to date to assess the effect of coffee in analyses that included a variety of glucose and insulin measures. Furthermore, as the first investigation of its kind in South America, findings from our study extend the apparent protective effect of coffee in relation to diabetes, observed solely in high income country and Asian contexts up until this point. Lastly, we believe this is the first study to examine the association between coffee and plasma glucose following a two-hour post-meal test among those with known diabetes.

Some limitations of this present study deserve mention. This study was cross-sectional, limiting interpretation with regards to causal direction of associations, although the exclusion of those with known diabetes at baseline from our primary analyses would limit the possibility of reverse causation as an explanation for our findings. The use of a food frequency questionnaire to assess dietary intake can often be associated with measurement error, although this is likely to be non-differential with regard to diabetes status, which would underestimate protective effects reported, if present. Owing to the large size of the ELSA-Brasil study, we were not able to assess insulin resistance and secretion using more sophisticated methods, and thus relied on the relatively more crude plasma insulin levels and HOMA measurements. However, validation studies have shown these measures to be relatively well correlated with more accurate measures of insulin resistance and secretion such as the hyperinsulinemic-euglycemic clamp [34]. Lastly, although attempts were made to statistically adjust for possible confounding variables, we cannot rule out the possibility or residual or unknown confounding in our analyses.

Our data add to the now overwhelming evidence that coffee intake may have metabolic effects with potential benefits to those at risk for diabetes and the metabolic syndrome [1]. Further exploration of both the addition of sugar or artificial sweeteners and the quantities typically added is needed to gain insight as to the potential antagonistic effects of these additives on diabetes risk. Additionally, as milk and other dairy products (e.g., creamer) are often added to coffee in different parts of the world, investigation into their possible effects on the coffee-diabetes relationship is warranted, given the suggestion that dairy products may protect against the development of diabetes [35].

## Conclusion

Middle-aged and elderly Brazilian participants who reported consuming coffee two or more times per day had lower odds of presenting newly diagnosed diabetes. This association appeared to be driven by mechanisms operating postprandially rather than in the fasting state. We did not find appreciable differences between those who reported consuming regular vs. decaffeinated coffee, or those who reported consuming their coffee with sugar, artificial sweetener vs. no additives. Thus, our findings support the hypothesis that habitual coffee consumption is part of a dietary pattern that is protective against type 2 diabetes.

## Supporting Information

S1 Table Adjusted* associations of frequency of coffee consumption per day with newly diagnosed diabetes, by type of coffee, addition of sweetener, and preparation method, from ELSA-Brasil (2008–2012). † P-value for the test of any association between coffee consumption and the outcome of interest. * Model 1: adjusted for sex, age (years), ELSA-Brasil center. Model 2: + race/color (white, pardo, black, asian/indigenous), education (high school or less, some university or more), education of mother (high school or less, some university or more), smoking status (current, former, never smoker), alcohol intake (user, former user, never user), leisure time physical activity level (engage in physical activity one time per week or less, engage in physical activity two or more times per week), hypertension, family history of diabetes, daily fruit consumption, daily vegetable consumption, dairy product intake (g/day), beef intake (g/day), white rice intake (g/day), soda intake (g/day), juice intake (g/day), tea intake (g/day), % kcal from fat. Model 3: + body mass index, waist-hip ratio, C-reactive protein. Model 4: + Magnesium. Further adjustment for insulin measures (fasting and 2-hour postload) for diabetes, fasting glucose, two-hour postload glucose, and HbA1c analyses.
(DOCX)

S2 Table Adjusted* associations of quantity (in cups) of coffee per day with newly diagnosed diabetes and intermediate hyperglycemia, from ELSA-Brasil (2008–2012) (N = 12586). ^a^ For IFG, IGT, HbA1c analyses, n = 11245 after exclusion of participants with newly diagnosed diabetes. † P-value for the test of any association between coffee consumption and the outcome of interest. * Model 1: adjusted for sex, age (years), ELSA-Brasil center. Model 2: + race/color (white, pardo, black, asian/indigenous), education (high school or less, some university or more), education of mother (high school or less, some university or more), smoking status (current, former, never smoker), alcohol intake (user, former user, never user), leisure time physical activity level (engage in physical activity one time per week or less, engage in physical activity two or more times per week), hypertension, family history of diabetes, daily fruit consumption, daily vegetable consumption, dairy product intake (g/day), beef intake (g/day), white rice intake (g/day), soda intake (g/day), juice intake (g/day), tea intake (g/day), % kcal from fat. Model 3: + body mass index, waist-hip ratio, C-reactive protein. Model 4: + Magnesium. Further adjustment for insulin measures (fasting and 2-hour postload) for diabetes, fasting glucose, two-hour postload glucose, and HbA1c analyses.
(DOCX)
