# Supplementary material for: Coffee Consumption, Newly Diagnosed Diabetes, and Other Alterations in Glucose Homeostasis: A Cross-Sectional Analysis of the Longitudinal Study of Adult Health (ELSA-Brasil)
Source: PLoS One. 2015 May 15;10(5):e0126469. doi: 10.1371/journal.pone.0126469 (PMC4433107; doi:10.1371/journal.pone.0126469)
Supplement: S1 Table — † P-value for the test of any association between coffee consumption and the outcome of interest. * Model 1: adjusted for sex, age (years), ELSA-Brasil center. Model 2: + race/color (white, pardo, black, asian/indigenous), education (high school or less, some university or more), education of mother (high school or less, some university or more), smoking status (current, former, never smoker), alcohol intake (user, former user, never user), leisure time physical activity level (engage in physical activity one time per week or less, engage in physical activity two or more times per week), hypertension, family history of diabetes, daily fruit consumption, daily vegetable consumption, dairy product intake (g/day), beef intake (g/day), white rice intake (g/day), soda intake (g/day), juice intake (g/day), tea intake (g/day), % kcal from fat. Model 3: + body mass index, waist-hip ratio, C-reactive protein. Model 4: + Magnesium. Further adjustment for insulin measures (fasting and 2-hour postload) for diabetes, fasting glucose, two-hour postload glucose, and HbA1c analyses. (DOCX) [file pone.0126469.s001.docx]

Table S1. Adjusted* associations of frequency of coffee consumption per day with newly diagnosed diabetes, by type of coffee, addition of sweetener, and preparation method, from ELSA-Brasil (2008-2012)

|  | Never/almost never | ≤1/day | 2-3/day | >3/day | P-value† |
| --- | --- | --- | --- | --- | --- |
|  | OR (95% CI) | OR (95% CI) | OR (95% CI) | OR (95% CI) |  |
| **Type of coffee** | | | | | |
| Caffeinated (n=11149) | | | | | |
| Model 1 | 1.00 | .87 (.71-1.07) | .79 (.64-.96) | .76 (.61-.95) | .05 |
| Model 2 | 1.00 | .84 (.68-1.03) | .78 (.63-.97) | .75 (.60-.94) | .07 |
| Model 3 | 1.00 | .81 (.65-1.01) | .79 (.64-.98) | .74 (.59-.93) | .08 |
| Model 4 | 1.00 | .81 (.65-1.01) | .78 (.63-.97) | .75 (.60-.95) | .09 |
| Decaffeinated (n=259) | | | | | |
| Model 1 | 1.00 | .82 (.52-1.30) | .93 (.53-1.62) | .49 (.17-1.41) | .51 |
| Model 2 | 1.00 | .76 (.47-1.23) | .94 (.53-1.69) | .44 (.15-1.32) | .37 |
| Model 3 | 1.00 | .77 (.47-1.26) | .98 (.54-1.77) | .44 (.15-1.35) | .40 |
| Model 4 | 1.00 | .77 (.47-1.27) | .96 (.53-1.75) | .47 (.15-1.44) | .45 |
| **Addition of sweetener** | | | | | |
| Sugar (n=6649) | | | | | |
| Model 1 | 1.00 | .96 (.73-1.26) | .83 (.63-1.09) | .80 (.60-1.06) | .17 |
| Model 2 | 1.00 | .91 (.69-1.20) | .81 (.61-1.06) | .76 (.57-1.02) | .16 |
| Model 3 | 1.00 | .90 (.68-1.19) | .80 (.60-1.07) | .75 (.56-1.02) | .18 |
| Model 4 | 1.00 | .90 (.68-1.20) | .80 (.60-1.07) | .79 (.59-1.08) | .31 |
| Artificial sweetener (n=3230) | | | | | |
| Model 1 | 1.00 | .82 (.60-1.14) | .79 (.58-1.09) | .67 (.48-.95) | .13 |
| Model 2 | 1.00 | .76 (.55-1.07) | .77 (.55-1.07) | .64 (.45-.92) | .11 |
| Model 3 | 1.00 | .74 (.53-1.05) | .78 (.56-1.10) | .64 (.44-.91) | .09 |
| Model 4 | 1.00 | .73 (.52-1.03) | .78 (.56-1.10) | .64 (.45-.92) | .10 |
| Unsweetened (n=1472) | | | | | |
| Model 1 | 1.00 | .79 (.59-1.07) | .85 (.63-1.14) | .74 (.54-1.01) | .26 |
| Model 2 | 1.00 | .76 (.55-1.03) | .83 (.62-1.13) | .72 (.52-.99) | .18 |
| Model 3 | 1.00 | .75 (.55-1.03) | .85 (.62-1.16) | .70 (.50-.97) | .12 |
| Model 4 | 1.00 | .75 (.54-1.03) | .85 (.62-1.17) | .71 (.51-1.00) | .15 |
| **Preparation method** | | | | | |
| Filtered (n=9449) | | | | | |
| Model 1 | 1.00 | .91 (.74-1.13) | .83 (.67-1.03) | .80 (.64-1.00) | .15 |
| Model 2 | 1.00 | .84 (.67-1.04) | .79 (.64-.99) | .75 (.60-.95) | .10 |
| Model 3 | 1.00 | .81 (.65-1.01) | .80 (.64-1.00) | .75 (.59-.95) | .11 |
| Model 4 | 1.00 | .81 (.65-1.02) | .79 (.63-.99) | .76 (.60-.97) | .14 |
| Espresso (n=528) | | | | | |
| Model 1 | 1.00 | .74 (.46-1.18) | .66 (.40-1.07) | .48 (.26-.91) | .06 |
| Model 2 | 1.00 | .82 (.50-1.35) | .77 (.46-1.30) | .64 (.33-1.26) | .50 |
| Model 3 | 1.00 | .89 (.54-1.47) | .87 (.51-1.48) | .65 (.32-1.28) | .65 |
| Model 4 | 1.00 | .86 (.52-1.43) | .86 (.50-1.47) | .65 (.32-1.30) | .64 |
| Moka Pot (n=476) | | | | | |
| Model 1 | 1.00 | .64 (.39-1.04) | .71 (.44-1.15) | .46 (.22-.97) | .06 |
| Model 2 | 1.00 | .67 (.40-1.13) | .87 (.52-1.44) | .52 (.24-1.14) | .22 |
| Model 3 | 1.00 | .69 (.41-1.17) | .96 (.57-1.60) | .53 (.24-1.18) | .27 |
| Model 4 | 1.00 | .66 (.39-1.13) | .95 (.57-1.61) | .53 (.23-1.19) | .24 |
| Instant (n=920) | | | | | |
| Model 1 | 1.00 | .73 (.51-1.04) | .59 (.39-.90) | .78 (.43-1.42) | .07 |
| Model 2 | 1.00 | .78 (.54-1.13) | .63 (.40-.97) | 1.05 (.56-1.96) | .16 |
| Model 3 | 1.00 | .79 (.54-1.16) | .67 (.43-1.05) | 1.00 (.52-1.89) | .31 |
| Model 4 | 1.00 | .79 (.54-1.16) | .68 (.44-1.08) | 1.00 (.53-1.92) | .33 |

† P-value for the test of any association between coffee consumption and the outcome of interest

* Model 1: adjusted for sex, age (years), ELSA-Brasil center.

Model 2: + race/color (white, pardo, black, asian/indigenous), education (high school or less, some university or more), education of mother (high school or less, some university or more), smoking status (current, former, never smoker), alcohol intake (user, former user, never user), leisure time physical activity level (engage in physical activity one time per week or less, engage in physical activity two or more times per week), hypertension, family history of diabetes, daily fruit consumption, daily vegetable consumption, dairy product intake (g/day), beef intake (g/day), white rice intake (g/day), soda intake (g/day), juice intake (g/day), tea intake (g/day), % kcal from fat

Model 3: + body mass index, waist-hip ratio, C-reactive protein.

Model 4: + Magnesium. Further adjustment for insulin measures (fasting and 2-hour postload) for diabetes, fasting glucose, two-hour postload glucose, and HbA1c analyses.
